# Prognostic disparities in young patients based on breast cancer subtype: A population-based study from the SEER database

**DOI:** 10.1097/MD.0000000000033416

**Published:** 2023-03-31

**Authors:** Bing Chen, Xiaojuan Zhang, Yi Liu, Chuandong Wang

**Affiliations:** a Department of Thyroid and Breast Surgery, Xiamen Humanity Hospital Fujian Medical University, Xiamen, China; b Department of Radiology, Xiamen Humanity Hospital Fujian Medical University, Xiamen, China; c Department of Endoscopy, National Cancer Center/National Clinical Research Center for Cancer/Cancer Hospital, Chinese Academy of Medical Science and Peking Union Medical College, Beijing, China.

**Keywords:** breast cancer, clinicopathological characteristics, SEER, survival, triple-negative breast cancer

## Abstract

Triple-negative breast cancer (TNBC) is associated with younger age and worse long-term survival. However, the characteristics and prognosis of different subtypes of breast cancer (BC) in young (<40 years) patients have not yet been elucidated. The present population-based study explored the clinical and pathological characteristics of young TNBC patients and investigated their long-term survival. We enrolled patients from the Surveillance, Epidemiology, and End Results database younger than 40 years of age with primary BC. Cases were defined as patients with TNBC (hormone receptor [HR]−/human epidermal growth factor receptor 2 [HER2]−), and controls were patients with other subtypes of BC (HR−/HER2+, HR+/HER2−, and HR+/HER2+). Demographic, pathological, and radiotherapy, chemotherapy, and surgery data were extracted and the overall survival was the primary endpoint. We enrolled 14,234 young patients with BC in the present study, of whom 2798 (19.7%) had TNBC and 11,436 (80.3%) had another BC subtype. A higher proportion of TNBC patients than non-TNBC patients had a more advanced tumor-node-metastasis stage (II–IV 80.5% vs 73.1%, *P* < .001), and smaller proportions underwent radiotherapy (50.0% vs 53.3%, *P* = .002) and surgery (91.8% vs 92.9%, *P* < .001). TNBC was associated with significantly lower 5-year survival rates than other subtypes among patients with regional node positivity (0, 1–3, 4–9, ≥10: 54.2% vs 57.7%, 44.2% vs 55.9%, 31.0% vs 52.0%, and 27.7% vs 38.8%, *P* < .001) and those with different lymph node ratios (low, intermediate, high: 50.9% vs 56.0%, 34.6% vs 53.6%, and 24.8% vs 44.8%, *P* < .001). Our research is the first to investigate the relevant characteristics of young TNBC patients in comparison with those of young non-TNBC patients based on the surveillance, epidemiology, and end results database. We found that young TNBC patients have a higher pathological stage and worse long-term survival than young patients with other BC subtypes. These findings have implications in identifying young patients with TNBC for aggressive therapy and further investigations should be performed to explore new multimodal treatments for such patients.

## 1. Introduction

Breast cancer (BC) is the most common form of cancer in women worldwide, and the prognosis is poor.^[1]^ Cancer is caused by many changes within organisms and in the environment.^[2]^ The DNA of the most vertebrates especially mammals is depleted in CpG dinucleotides.^[3]^ The remaining CpGs clustering in DNA regions is generally referred to as CpG islands (CGIs). There has been growing interest in CGI because they are enriched in the promoters of genes^[3]^ and by altering DNA methylation in CGIs, they play important roles in the regulation of gene expression and gene silencing in biological processes such as X-chromosome inactivation, imprinting, silencing of intragenomic parasites^[4]^ and considerably, would help to discover the epigenetic causes of cancer,^[5]^ hence the aim of this study is to evaluate clinical and pathological characteristics of BC patients with different gene expressions.

Triple-negative breast cancer (TNBC), which is estrogen receptor-negative, progesterone receptor-negative, and human epidermal growth factor receptor 2 (HER2)-negative, accounts for 15% to 20% of BC cases.^[6]^ TNBC is associated with young age, advanced stage, and worse long-term survival compared to other subtypes.^[7–9]^ Thike et al^[10]^ analyzed 653 patients with TNBC and found worse disease-free survival and overall survival (OS) in young patients with TNBC compared to older patients with TNBC. Numerous other studies have reported similar results regarding the long-term prognosis in young patients with TNBC.^[11,12]^ Although younger patients with BC are usually able to undergo more intensive therapy, better survival cannot be achieved because younger patients tend to have more aggressive disease.^[13]^ It is particularly important to study clinical and pathological characteristics of young patients with different subtypes of BC for better treatments.

Lymph node (LN) metastases play a vital role in BC development, especially in TNBC. And LN assessment is an effective prognostic indicator in patients with BC.^[14,15]^ The lymph node ratio (LNR), defined as the ratio of positive LNs to total excised LNs, has been shown to be a promising predictor for patients with BC. Moreover, some studies have shown that LNR is an independent factor for OS.^[16,17]^

Although age as a prognostic factor has been assessed in BC, there are few studies revealing the characteristics of different subtypes of young BC patients. In the current population-based study, we sought to explore the clinical and pathological characteristics of young TNBC patients and investigate their long-term survival.

## 2. Materials and Methods

### 2.1. Patient screening

The present analysis selected data from the 18 registries of the surveillance, epidemiology, and end results (SEER) program. We enrolled BC patients diagnosed after 2010 since the HER2 receptor subtype was not available in the SEER database until 2010. The inclusion criteria were as follows: Female primary BC patients diagnosed at younger than 40 years; Unilateral invasive BC; Record of hormone receptor (HR) status, including estrogen receptor and progesterone receptor status, and HER2 receptor status; Complete clinical and pathological information, including age, race, marital status, 6^th^ American Joint Committee on Cancer tumor stage,^[18]^ and number of positive regional lymph nodes; and OS of more than 3 months. Patients who did not meet these criteria or had incomplete data were excluded from the study. This study was approved by the Ethics Committee of Xiamen Humanity Hospital Fujian Medical University.

### 2.2. Study design

The study population consisted of young patients with BC diagnosed between January 2010 and December 2017. This study used a case-control design to investigate the clinical and pathological outcomes of young patients with TNBC. Cases were defined as patients with TNBC (HR−/HER2−). Controls included patients with non-TNBC (HR−/HER2+, HR+/HER2−, and HR+/HER2+). We extracted demographic, pathological, and radiotherapy, chemotherapy, and surgery data. The LNR was calculated as the number of positive LNs divided by the number of resected LNs. We define a higher pathological state as a worse differentiation (grade III–IV) and more advanced tumor-node-metastasis (TNM) stage (II–IV).^[12]^ Patients were divided into low-risk (≤0.20), intermediate-risk (0.20–0.65), and high-risk (>0.65) LNR groups. OS was the primary endpoint.

### 2.3. Statistical analyses

SPSS 22.0 statistical software was used for statistical analysis.^[19]^ The demographic, pathological, and radiotherapy, chemotherapy, and surgery data were compared using the chi-square test. Subgroup analyses were performed to investigate the characteristics of TNBC and non-TNBC patients associated with OS. OS rates were calculated using the Kaplan–Meier method. Prognostic factors associated with survival were estimated by univariate and multivariable Cox proportional hazard models. To avoid confounding bias (age, race, and marital status), propensity score matching was performed using a small caliper of 0.1. Statistical significance was set at *P* < .05.

## 3. Results

### 3.1. Baseline characteristics

**Figure 1.**
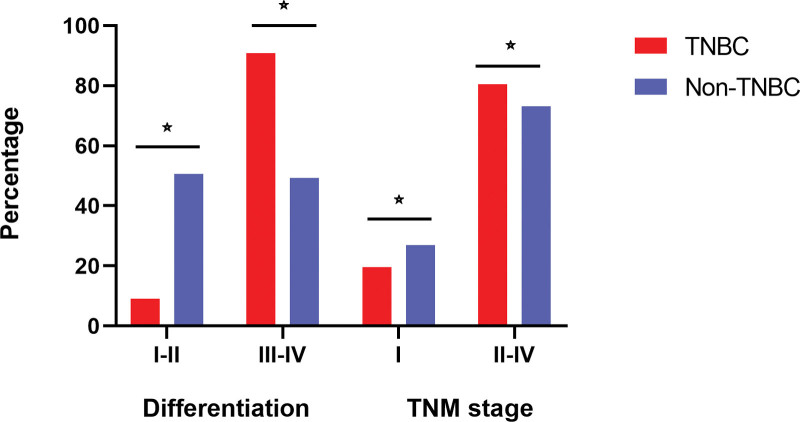
The pathological state of different subtype of BC.☆:*P* < .05. BC = breast cancer.

**Figure 2.**
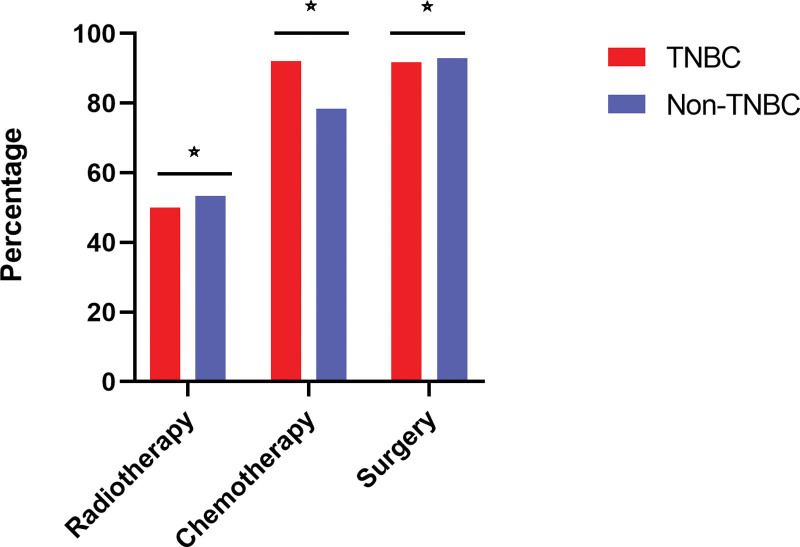
The anticancer therapy of different subtype of BC.☆:*P* < .05. BC = breast cancer.

We enrolled 14,234 young patients with BC in the present study, with 2798 (19.7%) patients having TNBC and 11,436 (80.3%) having non-TNBC. The major baseline characteristics of the study population are shown in Table 1. TNBC patients were less likely to be younger than 30 years (86.9% vs 89.1%, *P* = .002), white (68.8% vs 71.7%, *P <* .001) and married (57.5% vs 61.2%, *P <* .001) than patients with other subtypes. Further, TNBC patients were more likely to have a worse differentiation (grade III–IV, 90.9% vs 49.4%, *P* < .001) and later TNM stage (II–IV, 80.5% vs 73.1%, *P* < .001) than non-TNBC patients (Fig. 1), but they were less likely to have positive LNs (at least 1 positive node, 42.9% vs 53.6%, *P* < .001). Additionally, they were less likely to have undergone radiotherapy (50.0% vs 53.3%, *P* = .002) and surgery (91.8% vs 92.9%, *P <* .001) but more likely to have undergone chemotherapy (92.1% vs 78.4%, *P <* .001) (Fig. 2).

**Table 1:** Baseline characteristics of study population.

		TNBC	Non-TNBC	*P* value
Age	<20	1 (0.3%)	12 (0.1%)	.002
	20–29	365 (13.0%)	1237 (10.8%)	
	30–39	2432 (86.9%)	10,187 (89.1%)	
Race	White	1924 (68.8%)	8200 (71.7%)	<.001
	Black	597 (21.3%)	1622 (14.2%)	
	American Indian/Alaska Native	21 (0.8%)	83 (0.7%)	
	Asian or Pacific Islander	256 (9.1%)	1531 (13.4%)	
Marital status	Married	1609 (57.5%)	7002 (61.2%)	<.001
	Unmarried	1189 (42.5%)	4434 (38.8%)	
Laterality	Right-origin of primary	1422 (50.8%)	5724 (50.1%)	.407
	Left-origin of primary	1376 (49.2%)	5712 (49.9%)	
Receptor subtype	HR−/HER2−	2798 (100%)	0	<.001
	HR−/HER2+	0	1021 (8.9%)	
	HR+/HER2−	0	7755 (67.8%)	
	HR+/HER2+	0	2660 (23.3%)	
TNM stage	I	547 (19.5%)	3074 (26.9%)	<.001
	II	1583 (56.6%)	5344 (46.7%)	
	III	544 (19.4%)	2273 (19.9%)	
	IV	124 (4.4%)	745 (6.5%)	
Grade	Well differentiated; Grade I	15 (0.5%)	1116 (9.8%)	<.001
	Moderately differentiated; Grade II	239 (8.5%)	4677 (40.9%)	
	Poorly differentiated; Grade III	2519 (90.0%)	5598 (49.0%)	
	Undifferentiated differentiated; Grade IV	25 (0.9%)	45 (0.4%)	
Regional nodes positive	≥10	420 (15.0%)	1764 (15.4%)	<.001
	0	1598 (57.1%)	5309 (46.4%)	
	1–3	625 (22.3%)	3333 (29.1%)	
	4–9	155 (5.5%)	1030 (9.0%)	
Radiotherapy	No	1400 (50.0%)	5337 (46.7%)	.002
	Yes	1398 (50.0%)	6099 (53.3%)	
Chemotherapy	No	222 (7.9%)	2475 (21.6%)	<.001
	Yes	2576 (92.1%)	8961 (78.4%)	
Surgery	No	229 (8.2%)	814 (7.1%)	<.001
	Yes	2569 (91.8%)	10,622 (92.9%)	

HR = hormone receptor, HER2 = human epidermal growth factor receptor 2, TNM = tumor-node-metastasis, TNBC = triple-negative breast cancer.

### 3.2. Survival analysis

**Figure 3.**
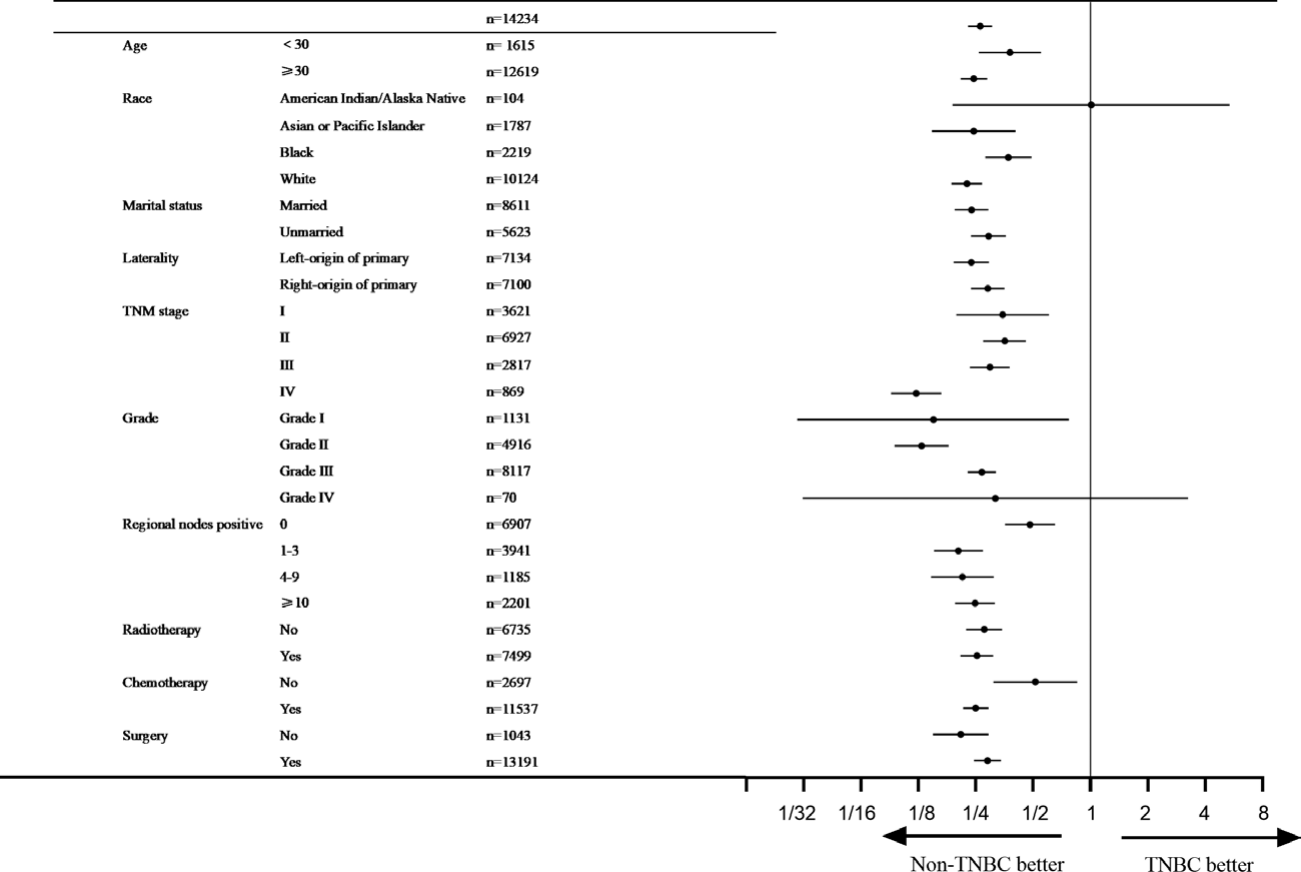
Hazard ratio for overall survival. Forest plot evaluating the impact of different subtype of BC. BC = breast cancer.

**Figure 4.**
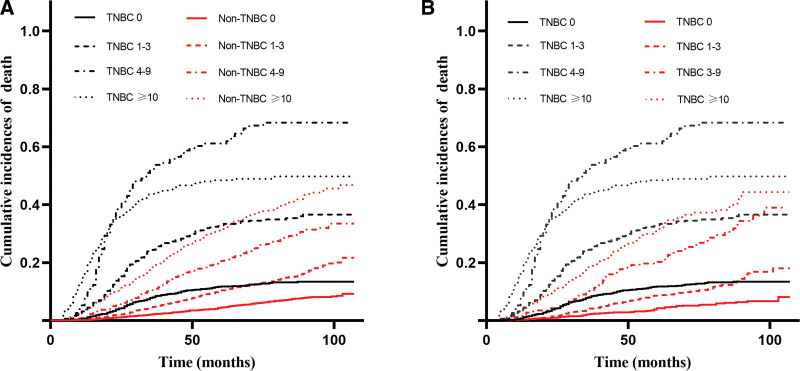
Overall survival according to positive regional nodes. (A) OS for patients with positive regional nodes. (B) OS for patients with positive regional nodes after propensity score matching. OS = overall survival.

**Figure 5.**
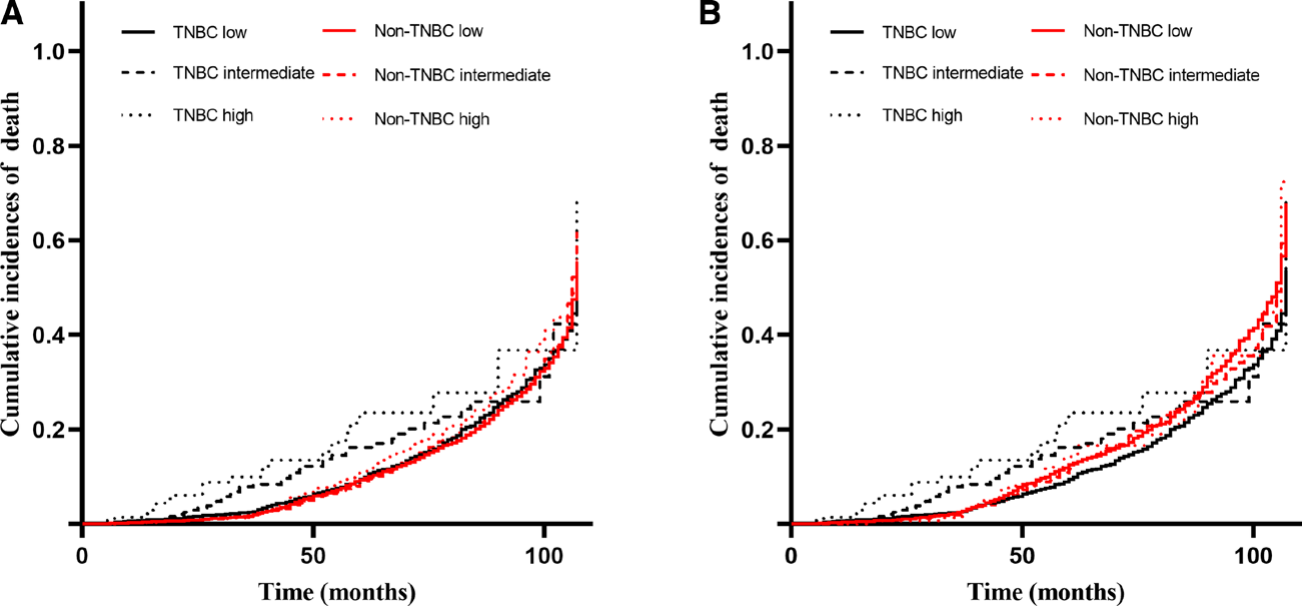
Overall survival according to LNR. (A) OS for patients with LNR. (B) OS for patients with LNR after propensity score matching. LNR = lymph node ratio, OS = overall survival.

Univariate and multivariate analyses confirmed that unmarried status, TNBC, higher TNM stage, higher grade, more positive regional nodes, and lack of surgery were associated with a worse prognosis (Table 2). To better understand the impact of BC subtype on OS, a subgroup analysis was performed. A forest plot revealed that TNBC was related to an increased risk of death in all groups except the American Indian/Alaska native and grade IV groups (Fig. 3). We compared the OS between the TNBC and non-TNBC groups based on positive regional nodes and LNR. Propensity score matching was used to avoid baseline bias. Significant differences in 5-year survival rates were found in patients with and without TNBC in the regional node-positive group (0, 1–3, 4–9, ≥10: 54.2% vs 57.7%, 44.2% vs 55.9%, 31.0% vs 52.0%, and 27.7% vs 38.8%, *P* < .001) and in different LNR groups (low, intermediate, high: 50.9% vs 56.0%, 34.6% vs 53.6%, and 24.8% vs 44.8%, *P* < .001) (Figs. 4 and 5). Survival analysis after propensity score matching also demonstrated that patients with TNBC had worse prognosis (*P* < .001).

**Table 2:** Univariate and multivariate analysis of overall survival.

Variable	Univariate analysis		Multivariate analysis	
	HR (95% CI)	*P* value	HR (95% CI)	*P* value
Age				
<20	References		References	
20–29	2.814 (0.395, 20.032)	.302	1.630 (0.228, 11.625)	.626
30–39	2.086 (0.294, 14.815)	.462	1.508 (0.212, 10.734)	.682
Race				
American Indian/Alaska Native	References		References	
Asian or Pacific Islander	0.759 (0.449, 1.283)	.303	0.711 (0.420, 1.204)	.205
Black	1.724 (1.032, 2.881)	.037	1.128 (0.673, 1.889)	.647
White	0.980 (0.589, 1.630)	.938	0.846 (0.508, 1.409)	.521
Marital status				
Married	References		References	
Unmarried	1.381 (1.270, 1.503)	<.001	1.155 (1.057, 1.263)	.001
Laterality				
Left-origin of primary	References		References	
Right-origin of primary	0.953 (0.877, 1.037)	.265	0.953 (0.877, 1.037)	.266
Receptor subtype				
HR-/HER2-	References		References	
HR-/HER2+	0.599 (0.504, 0.711)	<.001	0.359 (0.301, 0.429)	<.001
HR+/HER2-	0.495 (0.449, 0.545)	<.001	0.473 (0.424, 0.527)	<.001
HR+/HER2+	0.381 (0.332, 0.438)	<.001	0.264 (0.228, 0.305)	<.001
TNM stage				
I	References		References	
II	2.446 (2.062, 2.902)	<.001	1.513 (1.251, 1.828)	<.001
III	7.309 (6.168, 8.661)	<.001	3.558 (2.887, 4.385)	<.001
IV	22.158 (18.560, 26.452)	<.001	10.584 (8.497, 13.183)	<.001
Grade				
Grade I	References		References	
Grade II	2.249 (1.724, 2.935)	<.001	1.561 (1.193, 2.043)	.001
Grade III	3.936 (3.041, 5.094)	<.001	2.283 (1.747, 2.982)	<.001
Grade IV	5.532 (3.302, 9.267)	<.001	1.918 (1.137, 3.237)	.015
Regional nodes positive				
0	References		References	
1–3	2.310 (2.047, 2.607)	<.001	1.779 (1.552, 2.040)	<.001
4–9	4.751 (4.133, 5.462)	<.001	2.126 (1.782, 2.536)	<.001
≥10	6.768 (6.033, 7.594)	<.001	2.429 (2.085, 2.829)	<.001
Radiotherapy				
No	References		References	
Yes	1.164 (1.070, 1.267)	<.001	1.032 (0.939, 1.135)	.507
Chemotherapy				
No	References		References	
Yes	1.741 (1.532, 1.978)	<.001	0.924 (0.805, 1.062)	.266
Surgery				
No	References		References	
Yes	0.217 (0.196, 0.241)	<.001	0.728 (0.632, 0.840)	<.001

HR = hazard ratio, HER2 = human epidermal growth factor receptor 2, TNM = tumor-node-metastasis.

## 4. Discussion

TNBC is usually diagnosed at a young age.^[7,8,20,21]^ Numerous studies have demonstrated that BC patients younger than 40 years have a distinct disease with consistently poor long-term prognosis.^[22–26]^ However, there is little evidence regarding the clinical and pathological characteristics of young (<40 years) TNBC patients in comparison with those of non-TNBC patients. To the best of our knowledge, the present study is the first to investigate the relevant characteristics of young TNBC patients in comparison with those of young non-TNBC patients based on the SEER database. Our study revealed a markedly higher pathological stage and less relevant anticancer treatments, except chemotherapy, in TNBC patients, which are similar to the results of a previous study.^[12,27,28]^ Furthermore, multivariate and subgroup analyses revealed that TNBC was associated with a worse prognosis. Moreover, TNBC was associated with a worse OS than non-TNBC in all LNR groups. Young patients with TNBC are more likely to have a higher pathological stage and worse long-term survival.

This study involved young BC patients, 42.9% of TNBC patients had LN metastasis, which is higher than the rate reported in a previous study of TNBC.^[29]^ Young patients are more likely to have aggressive disease, which may be the result of a higher rate of BRCA mutations.^[30,31]^ However, unlike previous studies, our investigation found that fewer patients with TNBC had positive regional nodes than patients with other BC subtypes (42.9% vs 53.6%, *P* < .001). Additional chemotherapy in patients with TNBC may reduce the rate of positive LNs. Interestingly, TNBC patients were less likely than non-TNBC patients to undergo surgery, despite a higher rate of chemotherapy. It is not beneficial for patients in terms of the tumor loads.^[32]^

Because the patients enrolled in our study were young, we focused on the 5-year survival rate instead of the short-term prognosis. We found that TNBC patients had worse 5-year survival than non-TNBC patients among young BC patients, and the analysis after propensity score matching confirmed this finding (*P* < .001). Moreover, multivariate and subgroup analyses identified that TNBC was associated with an increased overall risk of death among young patients compared with non-TNBC. Although chemotherapy is now acknowledged as a systemic treatment for TNBC patients, we observed a limited effect. This may be due to a lack of molecular targets in these patients.^[12]^ Steward et al^[33]^ demonstrated that radiation therapy after lumpectomy is associated with better prognosis in patients with TNBC. Other studies have also confirmed that postmastectomy radiation therapy increases disease-free survival in patients with *T*1–*T*2 disease.^[34]^ However, the single treatment has limited effect on the long-term prognosis due to local or distant residual lesions.^[35]^ Our survival analysis also showed that LN metastasis decreased survival, which indicates that reduced residual tumor load plays a vital role in young patients with BC. Therefore, multimodality treatments should be encouraged, including chemotherapy, radiotherapy, and surgery, especially in young patients with TNBC. Further larger scale studies are needed for individualized therapies in such patients.

This study has some limitations. First, although propensity score matching was used to avoid baseline bias, treatments such as chemotherapy, radiotherapy, and surgery still influenced long-term survival. Second, some variables were not included, including chemotherapy regimen, surgical treatment, and gene mutations. Last, the lack of further classification of non-TNBC is not conductive to the expansion of research results.

## 5. Conclusion

Our results suggest that young patients with TNBC are more likely to have a higher pathological stage than other BC subtypes, which may lead to a worse long-term survival. As young patients tend to have better survival expectations, these findings also have implications in identifying young patients with TNBC for aggressive therapy. Large RCTs are needed to investigate better treatments and improve follow up in these patients.

## Acknowledgments

The authors thank all colleagues and nurses who provided care to the patients in this study.

## Author contributions

**Conceptualization:** Bing Chen, Xiaojuan Zhang, Yi Liu, Chuandong Wang.

**Data curation:** Bing Chen, Yi Liu, Chuandong Wang.

**Formal analysis:** Chuandong Wang.

**Funding acquisition:** Chuandong Wang.

**Investigation:** Chuandong Wang.

**Methodology:** Xiaojuan Zhang, Chuandong Wang.

**Project administration:** Xiaojuan Zhang, Chuandong Wang.

**Resources:** Xiaojuan Zhang.

**Software:** Bing Chen.

**Writing – original draft:** Bing Chen, Yi Liu, Chuandong Wang.

**Writing – review & editing:** Bing Chen, Yi Liu, Chuandong Wang.
